# Supplementary material for: From the outer space to the inner cell: deconvoluting the complexity of Bacillus subtilis disulfide stress responses by redox state and absolute abundance quantification of extracellular, membrane, and cytosolic proteins
Source: Microbiol Spectr. 2024 Feb 15;12(4):e02616-23. doi: 10.1128/spectrum.02616-23 (PMC10986503; doi:10.1128/spectrum.02616-23)
Supplement: Supplemental material — Table S1; Figures S1 to S7. [file spectrum.02616-23-s0001.docx]

Supplemental Material

From the outer space to the inner cell: Deconvoluting the complexity of *Bacillus subtilis* disulfide stress responses by redox-state and absolute abundance quantification of extracellular, membrane and cytosolic proteins

Borja Ferrero-Bordera^1^, Jürgen Bartel^1^, Jan Maarten van Dijl^2^, Dörte Becher^1^, Sandra Maaß^1#^

1. University of Greifswald, Centre of Functional Genomics of Microbes, Department of Microbial Proteomics, Institute of Microbiology, D-17489 Greifswald, Germany
2. University of Groningen, University Medical Center Groningen, Department of Medical Microbiology, Hanzeplein 1, P.O. Box 30001, 9700RB Groningen, The Netherlands

^#^ Correspondence: sandra.maass@uni-greifswald.de; Tel.: +49 (0)3834 420 5921

Supplemental Table 1. Concentration standards added to the culture supernatant including the physicochemical properties that were taken into consideration for their selection. The candidate proteins were also checked to not exhibit shared tryptic peptides with proteins from B. subtilis and the applied UPS2 standards. Concentration and amount are described for the stock solution of the concentration standard mix that was used for spiking the culture supernatant.

| Protein | Organism | Uniprot  Accession | Supplier | Chain length (amino acids) | MW (KDa) | pI | Gravy index | Concentration (ng/µl) | Protein amount (fmol) |
| --- | --- | --- | --- | --- | --- | --- | --- | --- | --- |
| alpha-Lactalbumin | Bos taurus (Bovine) | P00711 | Sigma Merck | 122 | 14 | 4.4 | -0.169 | 174.07 | **5000** |
| Glycerol-3-Phosphate  dehydrogenase | Oryctolagus cuniculus (Rabbit) | P46406 | Sigma Merck | 334 | 37 | 3.5 | -0.053 | 460.05 | **5000** |
| Alcohol  dehydrogenase | Saccharomyces cerevisiae (Yeast) | P00330 | Sigma Merck | 348 | 36.8 | 5.6 | 0.030 | 45.76 | **500** |
| Soybean trypsin inhibitor | Glycine may (Soybean) | P01071 | Gibco | 181 | 20 | 4.7 | -0.241 | 24.87 | **500** |
| Lisozyme | Gallus gallus (Chicken) | P00698 | Pierce | 129 | 14.3 | 11.4 | -0.150 | 17.78 | **500** |
| Bovine serum  albumin | Bos taurus (Bovine) | P02769 | Pierce | 582 | 69 | 4.8 | -0.433 | 8.60 | **50** |
| Carbonic anhydrase | Bos taurus (Bovine) | Q1LZA1 | Sigma Merck | 261 | 29 | 6.6 | -0.506 | 3.61 | **50** |


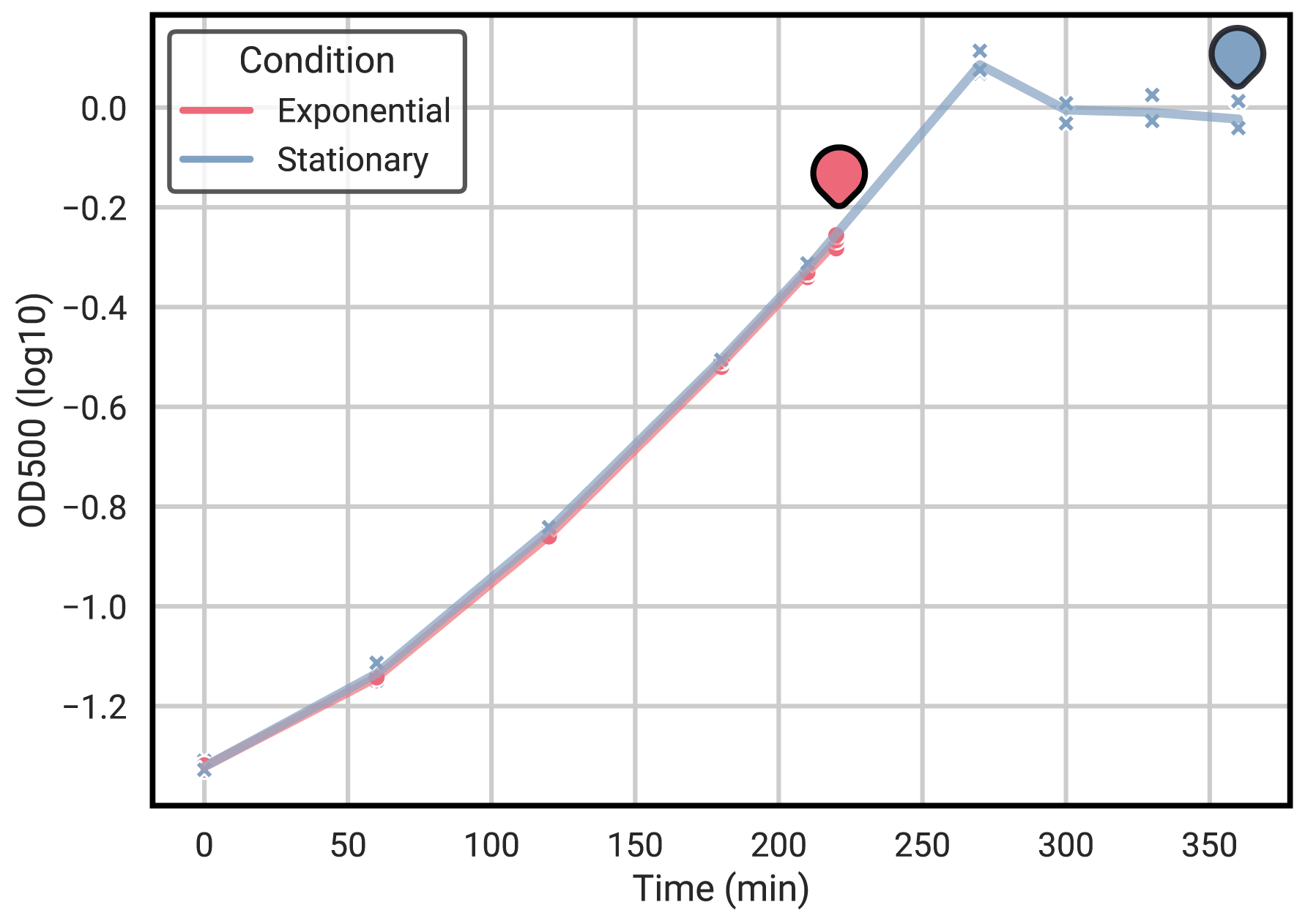


Supplemental Figure 1. B. subtilis cultivation in Belistky Minimal medium at 37 °C for extracellular protein absolute quantification. Samples were taken when the cultures reached OD_500_ = 0.4 (exponential) and two hours after the culture reached the maximal optical density (stationary).


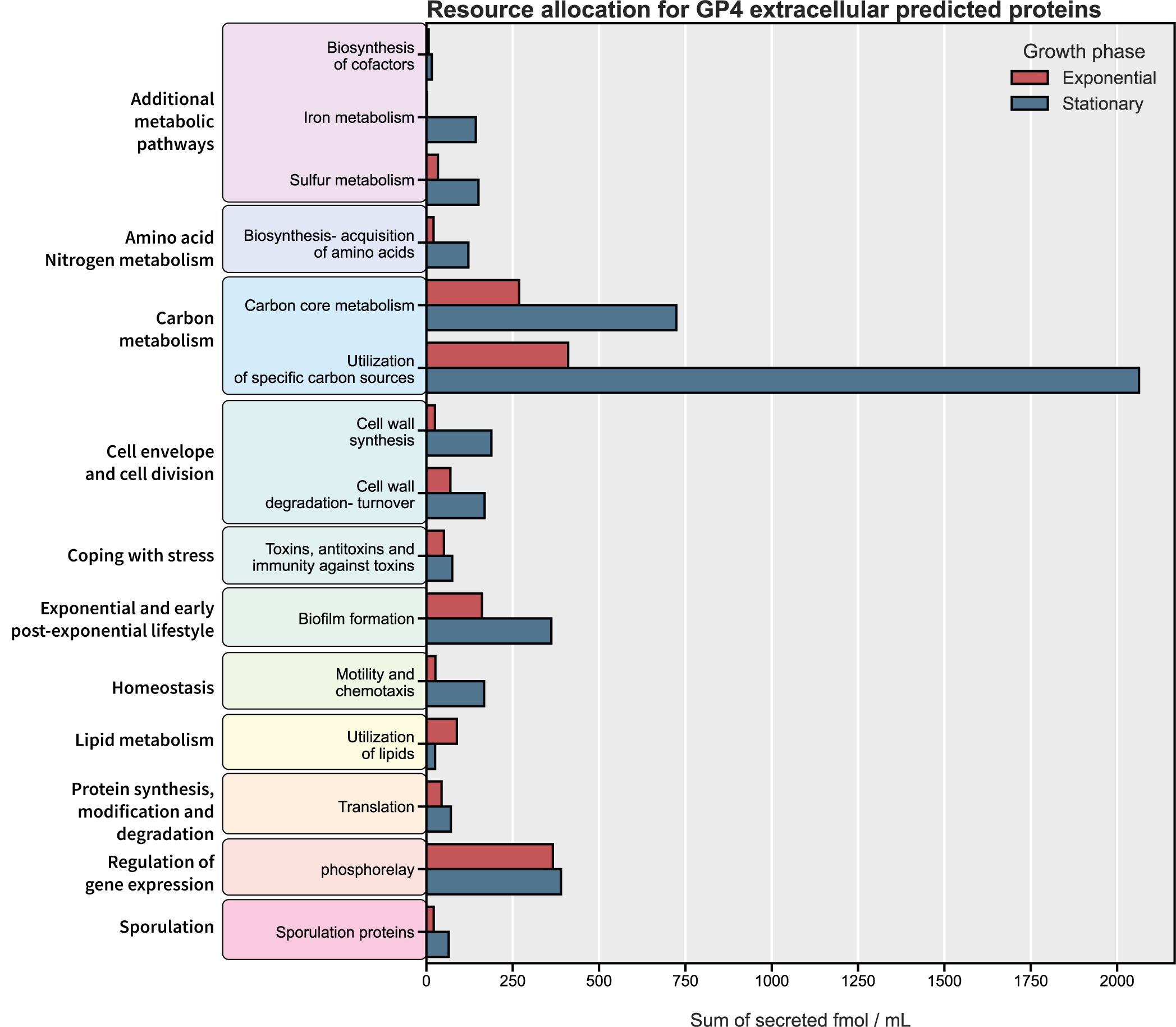


Supplemental Figure 2. Comparison of the sum of fmol per mL of secreted proteins quantified in exponential and stationary phase grouped by Subtiwiki functional categories. Only proteins with a predicted extracellular localization according to GP4 were considered.


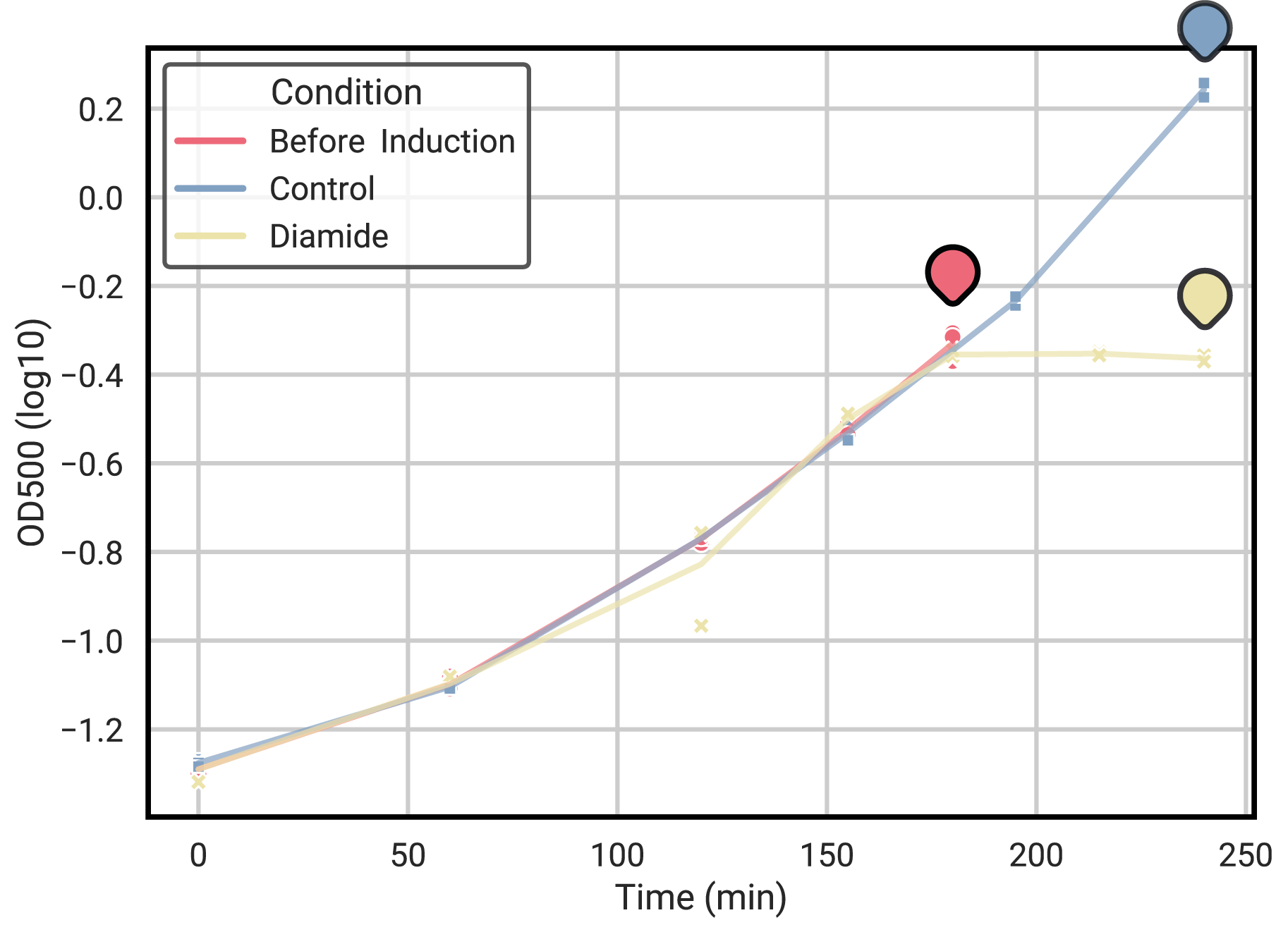


Supplemental Figure 3. B. subtilis cultivation in Belistky Minimal medium at 37 °C for cellular and extracellular protein absolute quantification under diamide stress. Diamide was added to a final concentration of 1 mM when the cultures reached OD_500_ = 0.4, referred as diamide samples. As reference, 2 non-treated conditions were assayed: one at the OD_500_ = 0.4 (before induction) and another one hour after the OD_500_ = 0.4 was reached (control). All conditions were assayed in 4 replicates.


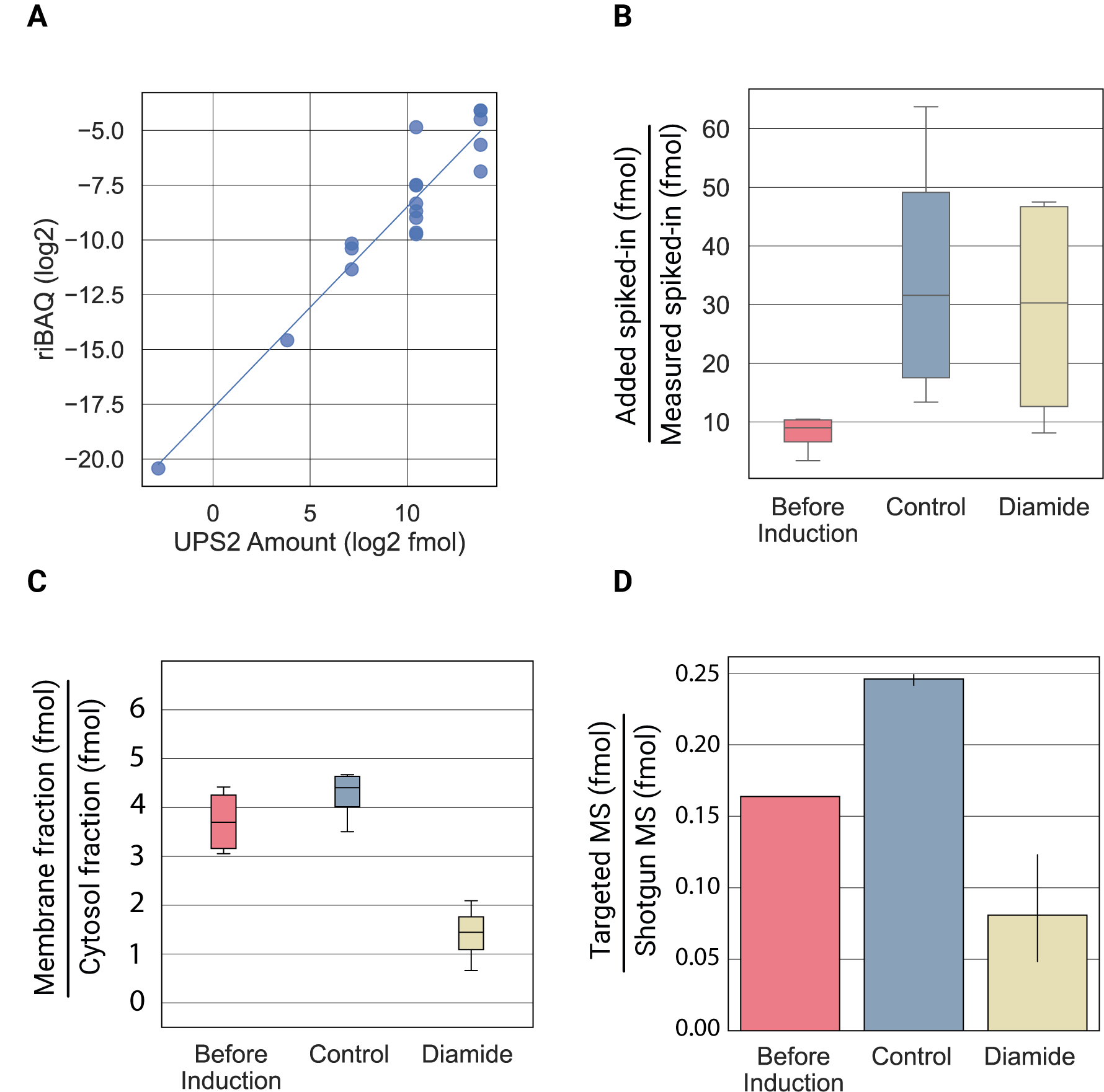


Supplemental Figure 4. A). UPS2 regression fitting for the disulfide stress experiment. B) Concentration factor for extracellular proteins in the disulfide stress experiment. C) Enrichment factor for membrane-enriched samples in the disulfide stress experiment. D) Correction factor for targeted MS and shotgun MS on the membrane fraction in the disulfide stress experiment.


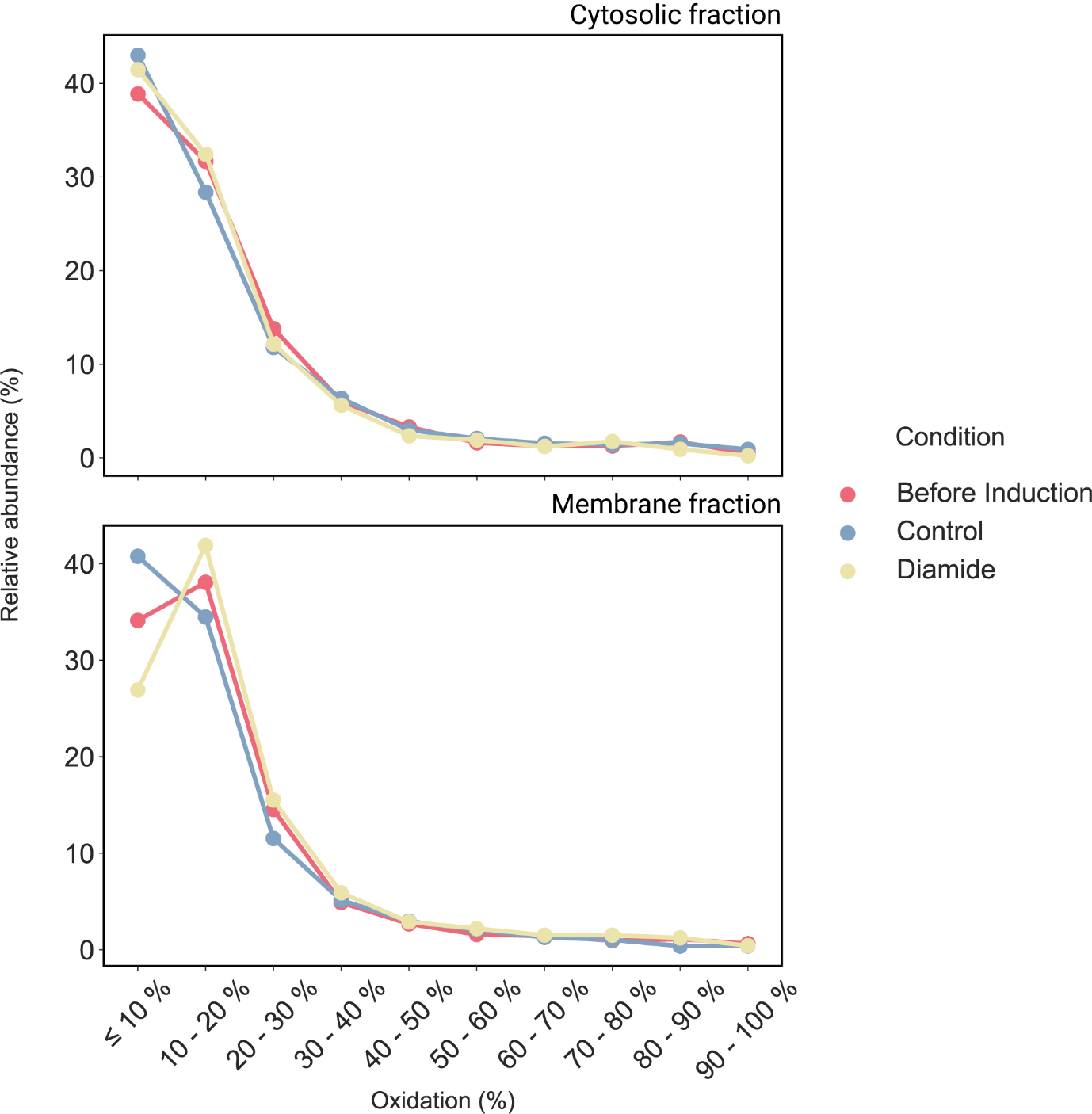


Supplemental Figure 5. Oxidation rates of one-cysteine peptides based on differential cysteine labeling measured for the cytosolic (top) and membrane (bottom) fractions in the disulfide stress experiment. The data points represent the relative abundance of peptides with oxidized cysteine residues per “bin” showing the degree to which their cysteine residues were oxidized. The oxidation rates are shown for samples obtained from control conditions, before induction and one hour after addition of 1 mM diamide to exponential growing cells (see Supplemental Figure 3).


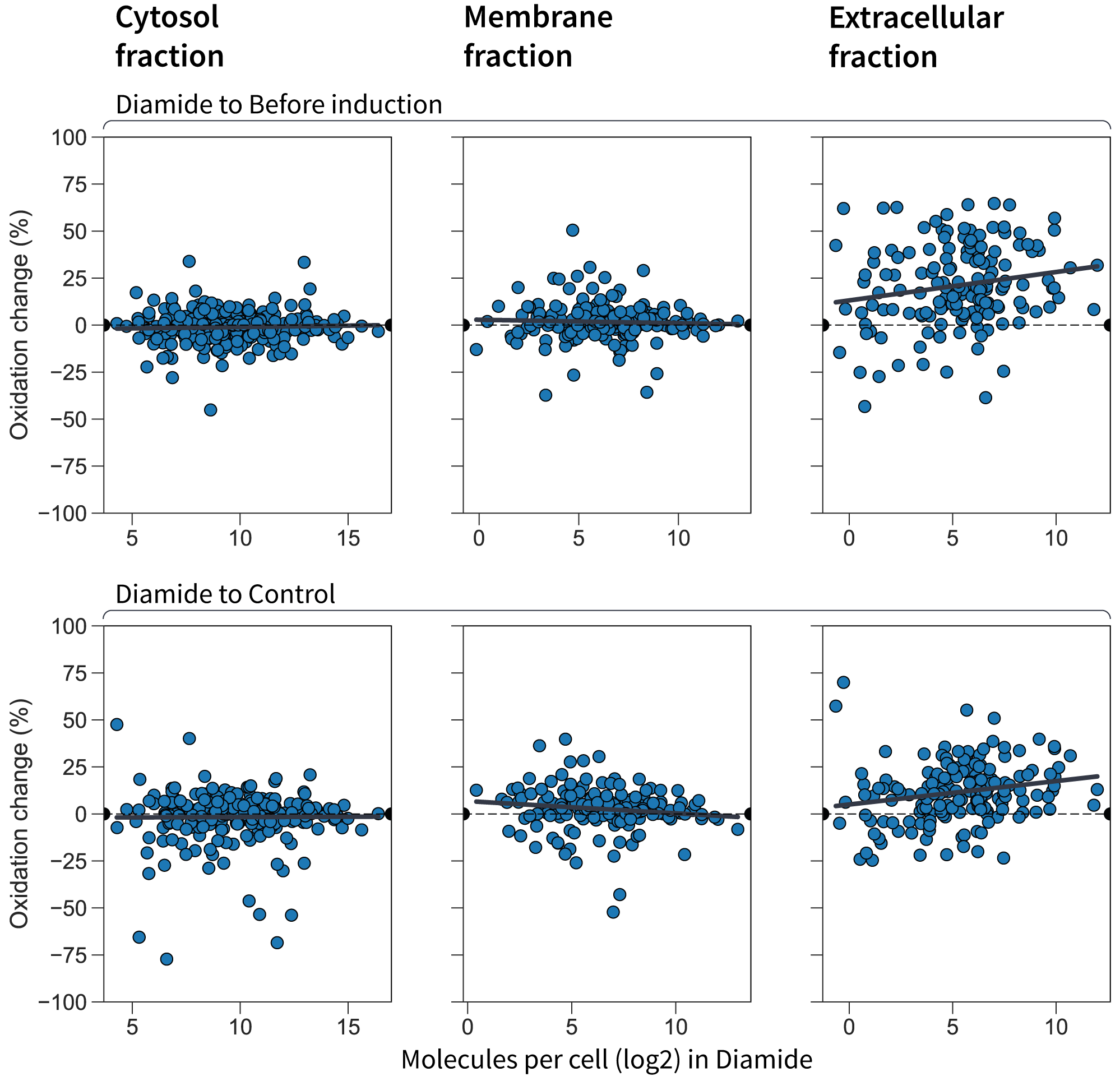


Supplemental Figure 6. Relationship between protein abundance and oxidation change in diamide samples relative to the sample before induction (top) and control samples (bottom) The correlation was made for proteins from the cytosolic (left), membrane (middle), and extracellular fraction (right).


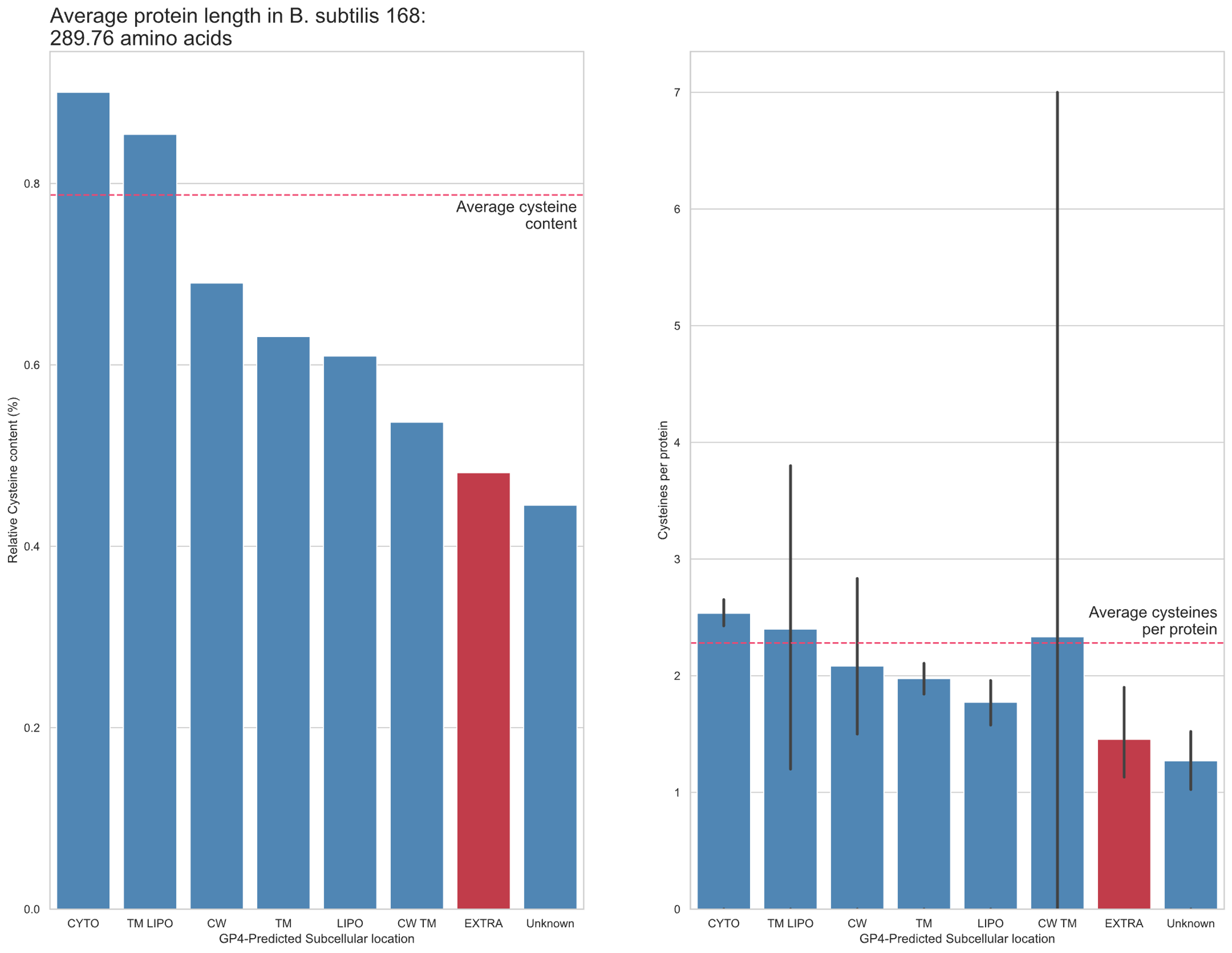


Supplemental Figure 7. Cysteine content (%) grouped by the predicted protein location (left). Average number of cysteines per protein grouped by predicted cellular location (right).
